# Kostenträgerrechnungen von strabologischen Operationen an einer Universitäts-Augenklinik

**DOI:** 10.1007/s00347-020-01227-x

**Published:** 2020-09-22

**Authors:** C. Framme, J. Gottschling, T. Kuiper, W. Lobbes, T. Palmaers, D. Brockmann, W. A. Lagrèze, K. Hufendiek

**Affiliations:** 1 Universitäts-Augenklinik, Medizinische Hochschule Hannover, Carl-Neuberg-Str. 1, 30652 Hannover, Deutschland; 2 Stabsstelle PM2 Klinische Leistungsentwicklung, Medizinische Hochschule Hannover, Carl-Neuberg-Str. 1, 30652 Hannover, Deutschland; 3 Stabsstelle PM2 OP-Management, Medizinische Hochschule Hannover, Carl-Neuberg-Str. 1, 30652 Hannover, Deutschland; 4 Klinik für Anästhesiologie und Intensivmedizin, Medizinische Hochschule Hannover, Carl-Neuberg-Str. 1, 30652 Hannover, Deutschland; 5 Klinik für Augenheilkunde, Medizinische Fakultät, Universitätsklinikum, Killianstr. 5, 79106 Freiburg, Deutschland

**Keywords:** Strabismuschirurgie, OP-Kosten, EBM, Ambulantes Operieren, Finanzierung, Strabismus surgery, Cost of surgery, Uniform evaluation benchmark, Financing, Outpatient surgery

## Abstract

**Hintergrund:**

Strabologische Operationen werden häufig in universitären Zentren durchgeführt. Ziel dieser Arbeit ist es, die Kosten dieser chirurgischen Leistung an einer Universitätsklinik zu ermitteln und die Kostenkompensation für den ambulanten Bereich zu überprüfen.

**Material und Methode:**

Von allen Strabismusoperationen an der Medizinischen Hochschule Hannover wurden in den Jahren 2018 und 2019 relevante OP-Daten wie Alter des Patienten, Anzahl der operierten Muskeln, Schnitt-Naht-Zeit, Präsenzzeiten der Chirurgen und Anästhesisten sowie der entsprechenden Funktionsdienste auf Basis des klinikeigenen Informationssystems evaluiert. Im Rahmen einer Kostenträgerrechnung wurden dabei die Kosten für Personal, Material, Raummiete und Overhead kalkuliert.

**Ergebnisse:**

Insgesamt wurden 302 Operationen (stationärer Anteil: 92,1 %) bis auf wenige Ausnahmen unter Vollnarkose durchgeführt. Das mittlere Alter der Patienten betrug 31 Jahre (Median: 26 Jahre), wobei 33 Patienten Kinder unter 6 Jahren waren. Im Mittel wurden 1,84 Muskeln pro Eingriff operiert. Die mittlere Schnitt-Naht-Zeit betrug 51,5 min, die mittlere Anästhesiezeit 85 min, die Präsenzzeit des Funktionsdienstes OP und auch die des Funktionsdienstes Anästhesie belief sich auf jeweils 104 min, die zusätzliche Aufwachraumzeit auf 66 min. Die am Gesamtprozess durchschnittlich entstandenen Personalkosten summierten sich auf 642,14 € zuzüglich durchschnittlich 109,23 € Material‑/Medikamentenkosten (Operation und Anästhesie) sowie Reinigung und Raumkosten (inklusive Infrastrukturkosten) von 178,71 €. Somit betrugen die Gesamtkosten einer durchschnittlichen strabologischen OP in unserem Kollektiv 930,08 € (Minimum: 491,01 €, Maximum: 1729,29 €). Kostenkalkulationen von Untergruppen ergaben aufgrund unterschiedlicher Behandlungszeiten (37 min für 1 Muskel bis 72 min für 3 und mehr Muskeln) respektive Anästhesiezeiten insbesondere bei Kindern <6 Jahren (durchschnittlich 22 min länger als bei Erwachsenen und Kindern >5 Jahren; Differenz 11 min [1 Muskel], 25 min [2 Muskeln] und 30 min [3 und mehr Muskeln]) deutlich höhere Kosten bei Kindernarkosen und einer höheren Anzahl operierter Muskeln. Das reine Kostenniveau einer Strabismusoperation an unserer Klinik erscheint durchschnittlich um den Faktor 2 höher als das Entgelt, welches für Strabismusoperationen über EBM (einheitlicher Bewertungsmaßstab) im ambulanten Bereich erlöst wird.

**Schlussfolgerungen:**

Die rein betriebswirtschaftlich errechneten Kosten für strabologische Operationen an einer Universitätsklinik sind signifikant höher als die im ambulanten Bereich nach Paragraf 115b, Absatz 1, SGB V aktuell zu erzielenden Erlöse. Unter diesen Bedingungen können solche Operationen ambulant nicht kostendeckend erbracht werden.

## Abstract (en)

**Background:**

Strabismus surgery is frequently carried out in university centers. The aim of this work was to calculate the costs of strabismus surgery at a university hospital and to assess the remuneration of costs for outpatient procedures.

**Material and methods:**

Of all strabismus surgeries at the Hanover Medical School in the years 2018 and 2019, relevant surgical data, such as patient age, number of muscles operated on, incision to suture time, attendance time of the surgeons and anesthetists as well as the nursing staff, were evaluated based on the clinics own information system. During this process, the costs for personnel, material, room rental charges and overheads were computed applying cost unit accounting.

**Results:**

A total of 302 operations (inpatient proportion 92.1%) were carried out in most cases with the patient under general anesthesia. The mean patient age was 31 years (median 26 years), with 33 patients being children under 6 years of age. On average 1.84 muscles were treated per intervention. The mean incision to suture time was 51.5 min, mean anesthesia time was 85 min, the attendance time of surgical as well as anesthesia nursing staff each accounted for 104 min, the additional time in the postanesthesia care unit added 66 min. Average personnel costs originating from the overall process amounted to 642.14 €, with the addition of 109.23 € for material and medication (surgery and anesthesia) and costs for cleaning and room rental (including overheads) of 178.71 €. Therefore, the overall costs of an average strabismus surgery in our collective added up to 930.08 € (minimum 491.01 €, maximum 1729.29 €). Cost accounting of subgroups yielded substantially higher costs for anesthesia in children as well as for higher numbers of muscles operated on due to different treatment duration (37 min for 1 muscle to 72 min for 3 muscles) and anesthesia time, especially in children <6 years of age (on average 22 min longer than adults and children >5 years; the differences being 11 min for 1 muscle, 25 min for 2 muscles and 30 min for 3 or more muscles). The pure costs of a strabismus surgery at this clinic seem on average to exceed the revenues for strabismus surgery in the outpatient sector calculated by the German uniform evaluation benchmark (EBM) by about a factor of 2.

**Conclusion:**

It could be shown that the purely economically calculated costs for strabismus surgery at a university clinic are significantly higher than the revenues achieved in the outpatient sector according to paragraph 115b, section 1, of the Social Security Act V (SGB V). Under these circumstances, such operations cannot be performed in a cost-effective manner.

Strabologische Operationen werden häufig in spezialisierten Zentren durchgeführt. Der Anteil pädiatrischer Fälle ist hierbei vergleichsweise hoch. Wie auch in anderen Subspezialitäten ist eine besondere Expertise sowohl auf diagnostischer als auch auf chirurgischer Ebene nötig, um die bestmögliche Qualität zu gewährleisten. Die moderne Augenheilkunde ist gerade in Universitätskliniken teilweise hoch spezialisiert in Subdisziplinen aufgeteilt, in denen Augenerkrankungen wie Katarakt, Glaukom, Glaskörper/Netzhaut, Orbita und Strabologie/Neuroophthalmologie von Spezialisten behandelt werden. Oftmals vermag der „Vorderabschnittsspezialist“ keine Operationen am Hinterabschnitt des Auges ausführen zu können und umgekehrt. Insbesondere der Bereich Orthoptik/Strabologie stellt hier nochmals ein besonderes Segment dar, da es vermutlich weniger strabologisch versierte Operateure gibt als Netzhautoperateure oder Kataraktoperateure. Strabologische Operationen sind eingebettet in eine oft sehr komplexe Diagnostik, und häufig ist eine langfristige postoperative Betreuung und Führung der Patienten z. B. in der Okklusionstherapie nötig.

Es ist allerdings auffällig, dass diese Spezialisierungen in den Vergütungssystemen unseres Gesundheitswesens finanziell nicht honoriert werden, sondern – zumindest in jüngerer Zeit – lediglich rein betriebswirtschaftlich die Kosten einer Leistung über Zeitdauer, Personal- und Materialkosten, Mieten, Geräte und Sonstiges summiert und pauschaliert vergütet werden. Dabei erscheinen viele Pauschalen allerdings deutlich unterfinanziert zu sein. Die speziellen Aspekte der Kindermedizin und ihrer Unterfinanzierung wurden 2019 in einem lesenswerten Beitrag im Deutschen Ärzteblatt diskutiert [20].

Die Augenheilkunde mag prädestiniert dafür sein, verschiedene Augenoperationen ambulant durchzuführen. Im Bereich der ambulanten Medizin gilt für die Vergütung der einheitliche Bewertungsmaßstab (EBM); [3]; für die stationäre Medizin wird über das DRG-System („Diagnosis Related Groups“); [11] abgerechnet. Beide Abrechnungssysteme beruhen auf pauschalierten Vergütungen mit entsprechend zugrunde liegenden Kostenkalkulationen [3, 9, 11]. Allerdings ist zu vermuten, dass den meisten ambulanten Pauschalen nur unzureichende betriebswirtschaftliche Berechnungen zugrunde liegen, was dazu führt, dass offensichtlich viele Leistungen gerade auch im ambulanten Bereich trotz fortgeführter Nachberechnungen zu niedrig bewertet werden.

Seit Einführung der DRGs in Deutschland ist die Krankenhausmedizin immer weiter betriebswirtschaftlich ausgerichtet worden. Liegezeiten verringerten sich über die Jahre deutlich, und gerade in der modernen Augenheilkunde erscheinen Liegezeiten von zum Teil nur noch 2 bis 3 Tagen postoperativ insbesondere für den Medizinischen Dienst der Krankenversicherung (MDK) [15] so niedrig, dass versucht wird, diese Zeiten noch weiter zu reduzieren und Leistungen aus Kostengründen so dann auch in den für die Krankenkassen finanziell günstigeren ambulanten Bereich zu verschieben.

Wenn denn schon auch ein Krankenhaus betriebswirtschaftlich so aufgestellt sein soll, dass es im Sinne einer „Schwarzen Null“ auskömmlich wirtschaften muss, liegt es auf der Hand, dass jede medizinische Leistung auch auskömmlich gegenfinanziert sein muss. Defizitäre Leistungen können aus rein betriebswirtschaftlichen Gründen dauerhaft nicht erbracht werden; dennoch müssen sie aber im Sinne der Patienten und des Versorgungsauftrages angeboten und erbracht werden, selbst wenn sie eben nicht auskömmlich sind. Dieses Dilemma ist vonseiten der Krankenhäuser nicht zu lösen.

Für den stationären Bereich existieren auf Basis der jährlichen InEK-Kalkulationen [11] für quasi jede Diagnose/Leistungsgruppe im Krankenhaus Kostenträgerrechnungen, welche sämtliche Kosten, wie oben bereits aufgezählt, anhand der Daten aus den Kalkulationshäusern darstellen und somit eine mittlere Preisfindung im Sinne der effektiven DRG-Pauschale ermöglichen. In der frühen Konvergenzphase des deutschen DRG-Systems ab dem Jahr 2003 („Lernendes System“) hat es auch in der Augenheilkunde bereits Abweichungen in den notwendigen Leistungserlösen gegeben [7, 14], die dem pauschalen Entgelt widersprachen und dann auch zu Nachkalkulationen und ggf. sogar DRG-Splits mit angepassten Neubepreisungen der Pauschalen geführt haben [4–6, 8, 18].

Die Abrechnung der ambulanten Eingriffe gegenüber der zuständigen Kassenärztlichen Vereinigung [3] erfolgt nach Bezifferung der abrechenbaren Leistungen aus dem gültigen EBM. Die Erlössumme pro Fall ergibt sich aus der mit der EBM-Ziffer hinterlegten Punktsumme für die Einzelleistung multipliziert mit dem von der Kassenärztlichen Bundesvereinigung (KBV) veröffentlichten Punktwert [3].

Ziel dieses Manuskriptes ist es nun, ungeachtet der medizinisch durchaus sinnvollen Diskussion über die Notwendigkeit strabologischer Operationen im stationären Setting zu evaluieren, ob die betriebswirtschaftlich berechenbare Leistung einer strabologischen Operation (Es wird hier prinzipiell lediglich die reine chirurgische OP-Leistung innerhalb des universitären Settings unabhängig vom stationären Aufenthalt betrachtet!) für das Krankenhaus ausreichend gegenfinanziert ist. Dabei gilt es zu bedenken, dass Augenkliniken (ggf. mit spezialisierten strabologisch-orthoptischen Abteilungen) regelhaft an größeren Krankenhäusern und Unikliniken vorgehalten werden, die ihrerseits einen deutlich höheren strukturell bedingten Fixkostenanteil („Overhead“-Kosten) haben als kleinere Einrichtungen und zusätzlich gerade auch Unikliniken die chirurgische Fortbildung von Fachärzten vorantreiben. Somit gehen in solchen Strukturen wahrscheinlich generell höhere Fixkosten für zu erbringende Leistungen ein, als dies ggf. in z. B. kleineren spezialisierten ambulanten chirurgischen Einrichtungen der Fall ist, in denen evtl. auch nicht in entsprechendem Maße aus-/weiter- und fortgebildet wird.

## Material und Methoden

In Zusammenarbeit mit administrativen Abteilungen der Medizinischen Hochschule Hannover (MHH) wurden die für die Wirtschaftlichkeitsberechnung erforderlichen Daten aus den im Hause verwendeten Datenbanken SAP [17] und COINS [2] extrahiert.

Die Schiel-OP-Patienten werden an unserem Haus in der Regel stationär und unter Vollnarkose chirurgisch versorgt. Nach fachärztlicher Überweisung werden die Patienten zunächst im Rahmen einer ambulanten Vorstellung in unserer Poliklinik (Abrechnung nach Hochschulambulanz-Pauschale) diagnostiziert. Ist eine operative Behandlung angezeigt, so wird in mindestens einer weiteren umfangreichen orthoptischen Untersuchung einschließlich Prismentrageversuch die Stabilität des Schielwinkels geprüft und ggf. eine chirurgische Intervention indiziert. Zu diesem Zeitpunkt erfolgt dann bereits auch die Aufklärung zur OP, und es wird der Patient regelhaft auch anästhesiologisch ambulant in der Prämedikationsambulanz der Klinik vorgestellt. Dieses ermöglicht die direkte stationäre Aufnahme am Operationstag mit zügiger Durchführung der OP.

Die MHH-Augenklinik benutzt täglich 2 Operationssäle (OP-Block III mit insgesamt 6 Sälen und jeweils 8 h Betriebszeit) über 5 Tage pro Woche für maßgeblich stationäre Patienten. Anästhesiologisch wird jeder Saal durch einen Anästhesisten sowie einen Funktionsdienst der Anästhesie betreut. Zusätzlich existiert ein beaufsichtigender oberärztlicher Anästhesist für 3 Säle. Für den Bereich Funktionsdienst OP werden 2 Pflegende (Instrumentierende[r] und Springer) benötigt. Der Operateur ist Oberarzt und wird von einem ärztlichen Kollegen (Kalkulationsbasis: 50 % Arzt in Weiterbildung; 50 % Facharzt für Augenheilkunde in chirurgischer Fortbildung) chirurgisch begleitet, wobei dieser im Rahmen der chirurgischen Fortbildung assistiert oder ebenfalls selbstständig und ggf. unter Aufsicht operiert. Zusätzlich wird der Aufwachraum von einem Funktionsdienst Anästhesie (Ansatz 25 % bei paralleler Betreuung von 4 Patienten) und einem erfahrenen Facharzt für Anästhesie partiell (Ansatz 10 %) betreut.

Strabologische Operationen werden maßgeblich an 2 vollen Tagen pro Woche jeweils in einem OP-Saal durchgeführt. Eingeschlossen wurden konsekutiv alle Operationen, bei denen aufgrund einer Schielstellung und/oder binokularer Doppelbilder eine strabologisch-chirurgische Intervention indiziert wurde. Für die Kostenberechnungen wurden die Mittelwerte der Schnitt-Naht-Zeiten (SNZ), die Anwesenheitszeiten der Operateure, der Anästhesisten und des jeweiligen Funktionsdienstes inklusive Rüstzeiten und Patientenvor- sowie Nachbereitung berechnet. Hinterlegt mit den jeweiligen Personalnormkosten für das Jahr 2020 (Tab. 1; [12]) wurden die Personalkosten pro einzelne Operation berechnet, und der entsprechende Durchschnittswert aller OPs wurde evaluiert. Die Personalnormkosten berücksichtigen die Arbeitgeberkosten (Arbeitgeber brutto) der MHH je Dienstart; inkludiert sind hier auch die tarifbedingten Jahressonderzahlungen und etwaige Zulagen sowie bereits bekannte Tarifsteigerungen; der Personalausfall von durchschnittlich 10 Tagen pro Jahr ist hier berücksichtigt durch Anrechnung einer Arbeitszeit von 20 Werktagen pro Monat (die Anzahl der Werktage in allen 2 berücksichtigten Jahren betrug jeweils *n* = 251); (Tab. 1; [12]). Inklusive der Berücksichtigung der Vorbereitungszeit der Operateure sowie der postoperativen Dokumentationszeit, die nicht vom System erfasst wird, aber pauschal mit 10 min präoperativ und 15 min postoperativ angesetzt wird, ergeben sich mit der zusätzlichen Überwachungszeit im Aufwachraum und dem kalkulierten „Overhead“ (Kosten für die nichtmedizinische und medizinische Infrastruktur – hier Personal) die Gesamtpersonalkosten pro Operation. Neben den Materialkosten für die OP selbst (Tab. 2; einmalig berechnet und fix für jede Operation zum Ansatz gebracht) beinhalten weitere Fixkosten die übrigen Sachkosten wie „chirurgische“ Medikamente, OP-Kleidung, Schuhe etc. sowie anästhesiologische Material- und Medikamentenkosten pro Patient. Zuzüglich der Raumkosten in Abhängigkeit der Nutzungsart (OP-Saal) und der Größe des Raumes (inklusive Overhead-Kosten hier für Gebäudeinfrastrukturen) errechnen sich schließlich die Gesamtkosten pro durchgeführte OP (Kostenträgerrechnung). Mitberücksichtigt in unserem Kollektiv sind partiell auch Lernkurven der Kollegen in chirurgischer Weiterbildung, insbesondere auch für die schonendere MISS-Technik („minimally invasive strabismus surgery“) mit limbusfernen Bindehauteröffnungen als Kleinschnitt, die zumindest in der Anfangsphase eines Lernenden Extrazeit kosten.

**Table 1:** 

Personalkosten	TG	Normkosten Personal 2020 (€)	Personalkosten/h (€)	Personalkosten/min (€)	Faktor (%)
Oberarzt Augenheilkunde	AE33	144.696,00	83,62	1,39	100
Facharzt Augenheilkunde	AE21	101.232,00	58,50	0,98	50
Assistenzarzt Augenheilkunde	AE13	84.252,00	48,69	0,81	50
Oberarzt Anästhesie Aufsicht	AE33	144.696,00	83,62	1,39	33
Facharzt Anästhesie	AE21	101.232,00	58,50	0,98	100
Funktionsdienst OP	EG9A	65.292,00	39,69	0,66	200
Funktionsdienst Anästhesie	EG9A	65.292,00	39,69	0,66	100
Facharzt Anästhesie Aufwachraum	AE23	117.108,00	67,68	1,13	10
Funktionsdienst Anästhesie Aufwachraum	EG9A	65.292,00	39,69	0,66	25

**Table 2:** 

Personalkosten	Summe (€)	Mittelwert (€)
OA-AUG	21.618,79	71,59
25 min OA-AUG Vor‑/Nachbereitung	10.494,50	34,75
50 % FA-AUG + 50 % ASS-AUG SNZ	13.920,01	46,09
15 min FA/AA-AUG Vor‑/Nachbereitung	3861,83	12,79
OP-Pflege (Vor‑/Ende Nachbereitung)	41.644,68	137,90
ANE-Pflege (Präsenzzeit)	20.823,23	68,95
AWR-ANE-Pflege + 10 min Vor- und Nachbereitung	5246,55	17,37
OA ANE Supervision (33 %)	14.618,33	48,41
FA ANE AWR (10 %)	2228,05	7,38
ANE-FA (Präsenz + Prämed)	37.722,50	124,91

Zu vermutende Unterschiede in der OP-Dauer und somit auch auf die Kosten können sich in Bezug auf einerseits die Anzahl der zu operierenden Augenmuskeln als andererseits auch auf das Alter der Patienten ergeben. So mag davon auszugehen sein, dass Kinder unter 6 Jahren ggf. eine längere anästhesiologische Vor- und Nachbereitungszeit benötigen als Erwachsene oder ältere Kinder. Auch für diese Untergruppen erfolgt die Kostenkalkulation.

Durch die duale Finanzierung der Krankenhäuser (Gebäude und Großgeräte finanziert über das Land Niedersachsen, Kosten der Patientenversorgung und Instandhaltung über die Kostenträger der Krankenversicherungen bzw. die Patienten selbst finanziert) brauchen in unserer Kalkulation gesamthaft die Kosten für bauliche Maßnahmen und primäre Anschaffung von Großgeräten nicht berücksichtigt zu werden.

## Ergebnisse

In den beiden Jahren 2018 und 2019 wurden insgesamt 302 Strabismusoperationen an der Augenklinik der Medizinischen Hochschule Hannover durchgeführt. Von diesen wurden 278 Operationen stationär und 24 Operationen „ambulant“ durchgeführt (stationäre Quote 92,1 %; allerdings wurden die meisten der 24 Fälle durch den MDK abrechnungstechnisch rückwirkend von stationär auf ambulant geändert). In nahezu allen Fällen wurde die Operation in Vollnarkose (*n* = 299) durchgeführt. Das mittlere Alter der Patienten betrug 31 Jahre (Median: 26 Jahre); 33 Patienten waren Kinder unter 6 Jahren (führt stationär in die DRG C10B: Eingriffe an den Augenmuskeln ohne erhöhten Aufwand <6 Jahren), 269 Patienten waren Erwachsene und Kinder in einem Alter über 5 Jahre (führt stationär in die DRG C10C: Eingriffe an den Augenmuskeln ohne erhöhten Aufwand >5 Jahre), wobei die Anzahl von Kindern zwischen 6 und 16 Jahren *n* = 78 und die von Patienten ab 17 Jahren *n* = 186 betrug. Die DRG C10A (Eingriffe an den Augenmuskeln mit erhöhtem Aufwand) wurde lediglich 3‑mal gruppiert.

Im Gesamtkollektiv wurden im Mittel 1,84 Muskeln pro Operation behandelt (1 Muskel: *n* = 77; 2 Muskeln: *n* = 196; 3 oder mehr Muskeln: *n* = 29). Für das Gesamtkollektiv betrug die mittlere Schnitt-Naht-Zeit 51,5 min, die Präsenzzeit der Pflege als auch die der Anästhesie 104 min und die zusätzliche Aufwachraumzeit 66 min.

Basierend auf Tab. 1 ergaben sich für das Gesamtkollektiv folgende Personalkosten pro durchgeführter Operation: Der Oberarzt Augenheilkunde (AE33-Kosten 1,39 €/min) kostet bei 51 min OP-Dauer plus 25 min perioperative Rüstzeit (gesamt 76 min) 106,34 €; der zweite Operateur (Mischkalkulation für 50 % Facharzt AE21 [0,98 €/min] und 50 % Assistenzarzt AE13 [0,81 €/Minute]) ist während der kompletten Operation zuzüglich 15 min Vor- und Nachbereitung anwesend und kostet somit durchschnittlich 58,88 €.

Für den Funktionsdienst OP (*n* = 2) ergibt sich für die OP-Vor- und Nachbereitung, die direkte Patientenvorbereitung und die postoperativen Arbeiten eine Anwesenheitszeit von durchschnittlich 104 min. Bei Normkosten von 0,66 €/min (EG9A) ergeben sich Kosten von 137,28 €. Auch für den Funktionsdienst Anästhesie (*n* = 1); (EG9A) ergeben sich 104 min für die OP-Präsenz inklusive Vor- und Nachbereitung (Kosten: 68,95 €) sowie nochmals 66 min für den Aufwachraum. (Hier werden nur 16,5 min angerechnet, da eine Pflegekraft bis zu 4 Patienten parallel versorgt zuzüglich 10 min Vor- und Nachbereitungszeit am Patienten, was somit einer anzurechnenden Gesamtzeit von 26,5 min entspricht.) Die Kosten für die Pflegearbeit im Aufwachraum liegen somit bei 17,37 €. Schließlich kostet der anästhesiologische Facharzt (AE21: 0,98 €/min) für die Dauer der Präsenz insgesamt 124,91 € (inklusive durchschnittlich 23 min in der Prämedikationsambulanz: 22,54 €). Hinzu kommt die oberärztliche Supervision im OP-Block (3 Säle), die bei einer AE33-Stelle (Kosten: 1,39 €/min) mit 33 % für die Dauer der Anästhesiepräsenz angerechnet wird (Kosten: 48,81 €). Zuletzt wird ein anästhesiologischer Facharzt (AE23-Kosten: 1,13 €/min) mit Ansatz 10 % für den Aufwachraum (entspricht etwa 7 min bei durchschnittlich 66 min Aufwachraumzeit) benötigt; hier ergeben sich Kosten von 7,38 €. Die Personalkosten summieren sich somit auf 570,13 €, und unter zusätzlicher Berücksichtigung der personalbedingten Infrastrukturkosten ergeben sich Personalgesamtkosten von 642,14 € für die durchschnittliche Strabismusoperation in unserem Kollektiv (Tab. 2).

Bezüglich der Sachkosten fallen pro Operation auf Basis der Berechnungsgrundlage laut Tab. 3 Kosten von 49,81 € an. Für Kittel, Haube, Mundschutz, OP-Schuhe, Verband und ophthalmologische Medikamente addieren sich durchschnittlich 18,59 €, die anästhesiologischen Materialkosten inklusive Medikamente belaufen sich durchschnittlich auf 40,83 € pro OP (und variieren nach Dauer der Narkose), sodass insgesamt 109,23 € an Materialkosten pro OP anfallen.

**Table 3:** 

Material	Preis (€)
Augen-Set	15,72
Pro Ophta Tupfer	0,65
Kompressen	0,60
Naht Marlin 6/0 2 × 45	11,18
Naht Marlin 7/0 2 × 45	6,58
Naht Polyester 5/0	7,87
Naht Marlin 9/0	7,21
*Summe*	*49,81*

Unter Berücksichtigung der Saalgröße, des Nebenraumprogramms und der Nutzungsart des Operationssaales (Augen-OP-Saal, Raum[luft]klasse 1b, 44 m^2^); [13] werden für die genutzten OP-Flächen 192.736 € pro Jahr veranschlagt. (Im MHH-Flächenbuch existieren Raumlisten inklusive der Quadratmeterangaben; die Grundlage zur Bewertung der genutzten Fläche bildet die jährlich aktualisierte Preisliste für sämtliche Flächen – orientiert an der DIN-NORM 277 – der MHH, die neben der Berücksichtigung von Mietnebenkosten auch die Kostenarten Reinigung, Energie, Technik, Ver- und Entsorgung sowie Objektbetreuung in der Kalkulation der Basispauschalen berücksichtigt.)

Auf Basis von 250 Werktagen pro Jahr liegen die Kosten bei 770,94 € pro Tag, was bei entsprechender Betriebszeit zu Minutenkosten von 1,61 € führt. Unter Berücksichtigung einer durchschnittlichen Saalnutzung pro Eingriff von ca. 111 min (inklusive Reinigung von 7 min) ergeben sich Infrastrukturkosten in Höhe von 178,71 € je Eingriff.

Die Gesamtkosten für die reine durchschnittliche OP-Leistung belaufen sich somit unter Addition der 3 Faktoren Personal, Material und Infrastruktur auf 930,08 €.

**Figure 1:**
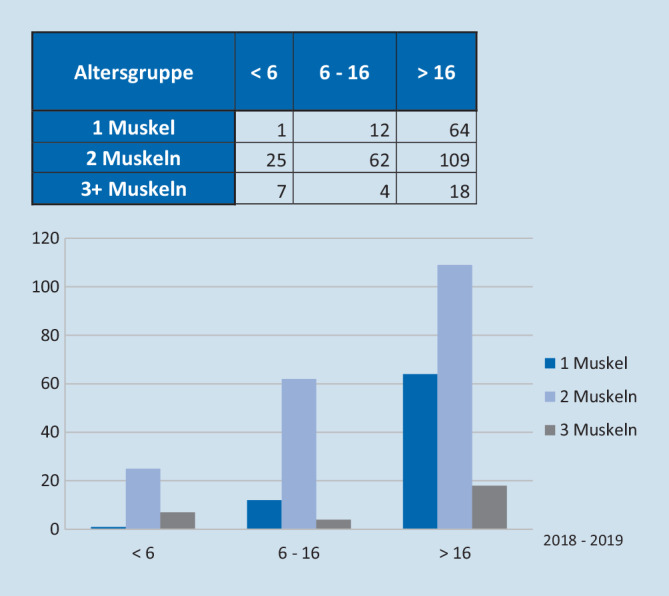

**Figure 2:**
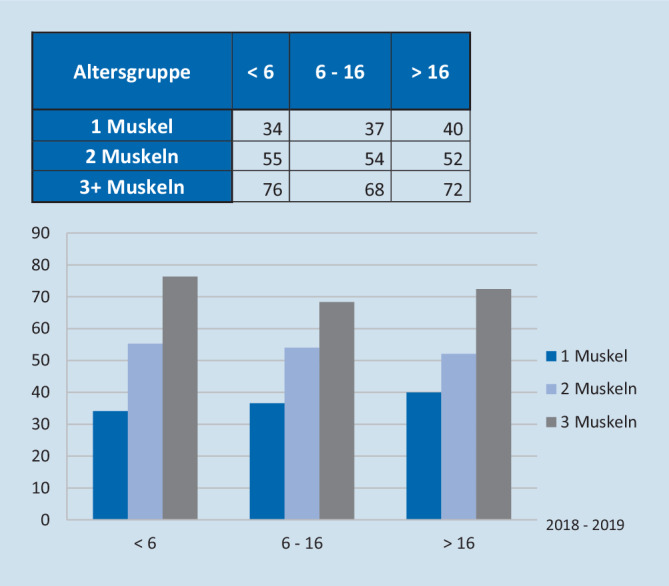

**Figure 3:**
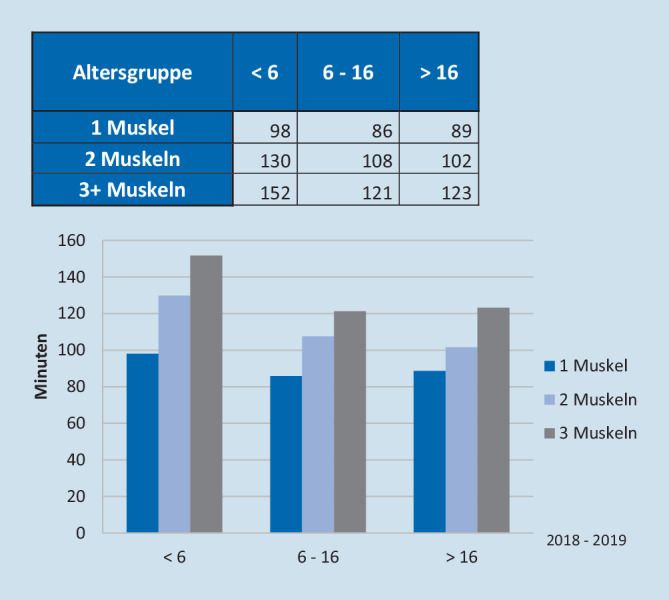

Da Personalkosten in der Regel den wesentlichen Anteil der Gesamtkosten darstellen, spielen die OP- und Anästhesiedauer eine erhebliche Rolle. Ein Split des Kollektives nach Anzahl operierter Muskeln pro Operation sowie nach Alter des Patienten ergab folgende Ergebnisse bezüglich der OP-Zeiten: Nahezu zwei Drittel aller Patienten waren mindestens 17 Jahre alt, und es wurden in der Mehrzahl der Fälle 2 Muskeln pro OP behandelt (Abb. 1). Unabhängig vom Patientenalter resultierten daraus in Abhängigkeit der Anzahl operierter Muskeln pro Eingriff längere OP-Zeiten (37 min für 1 Muskel, 54 min für 2 Muskeln und 72 min für 3 und mehr Muskeln) (Abb. 2). Bezüglich der Anästhesiezeiten zeigten sich insbesondere bei Kindern <6 Jahren deutlich längere Präsenzzeiten. Diese waren durchschnittlich 22 min länger als bei Patienten >5 Jahren. Es ergaben sich Differenzen von 11 min (1 Muskel), 25 min (2 Muskeln) und 30 min (3 und mehr Muskeln) und somit deutlich höhere Kosten in Abhängigkeit dieser Faktoren (Abb. 3). Pauschal konnte man mit einer durchschnittlichen Erhöhung der Kosten von 7,80 € pro zusätzlicher OP-Minute in unserem Kollektiv rechnen.

**Figure 4:**
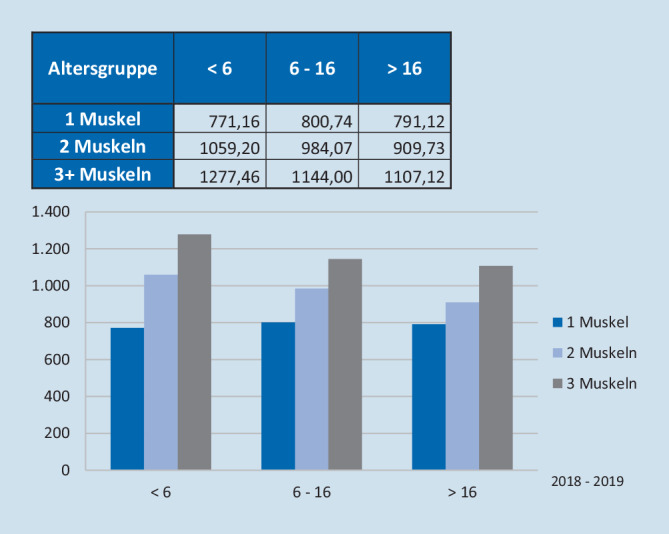

Die Abb. 4 gibt nun die Gesamtkosten der Operation in einer Matrix für die Untergruppierungen Alter und Muskelanzahl wieder (Abb. 4). Es zeigt sich, dass Operationen bei Kindern unter 6 Jahren (Mittelwert: 1096,77 €) deutlich teurer sind als bei Kindern zwischen 6 und 16 Jahren (Mittelwert: 964,07 €) und Erwachsenen über 16 Jahren (Mittelwert: 888,59 €). Ein ähnliches Gefälle ergibt sich nach Anzahl der operierten Muskeln mit durchschnittlich 1153,33 € (3 Muskeln), 952,31 € (2 Muskeln) und 792,36 € (1 Muskel). Operationen an 3 oder mehr Muskeln bei Kindern unter 6 Jahre sind mit im Mittel 1277,46 € die teuersten strabologischen Eingriffe (Abb. 4).

Das Kostenniveau für die reine Strabismus-OP (ohne stationäre Kosten!) an unserem Haus erscheint damit durchschnittlich um ungefähr den Faktor 2 höher als das Entgelt, welches für diese Operationen im ambulanten Bereich nach EBM mit Entgelten von 408,50 bis 662,80 € [3] inklusive der anästhesiologischen Leistung erlöst wird (s. Diskussion).

## Diskussion

Die Medizin in Deutschland steht verstärkt im Spannungsverhältnis zwischen Ökonomie und medizinischer Qualität. Dieser Sachverhalt führt dazu, dass medizinische Leistungen so günstig wie möglich angeboten werden müssen, um Kosten in Krankenhäusern und bei den Krankenkassen einzusparen. Überprüft wird diese möglichst günstigste Erbringung durch den MDK [15]. Primäre und sekundäre Fehlbelegungen im Krankenhaus werden entsprechend geahndet und erbrachte („Fehl“-)Leistungen nicht nur nicht adäquat bezahlt, sondern ab 2021 (für das Jahr 2020 ausgesetzt wegen COVID-19) sogar mit Strafzahlungen belegt.

Gerade in der modernen Augenheilkunde scheinen sich die Grenzen zwischen ambulanter und stationärer Leistungserbringung aufgrund der im Vergleich zu anderen Fachgebieten relativ kurzen Liegezeiten im Krankenhaus immer weiter anzunähern. Beispielhaft erscheint die extraokulare Strabismuschirurgie als ein Segment, für welches es Argumente für und wider stationäre Leistungserbringung gibt. Diese Diskussion sollte aber sicherlich jeweils personalisiert für den einzelnen Patienten geführt werden. Unabhängig von dieser inhaltlichen Diskussion ergibt sich ein wesentliches Problem für Krankenhäuser, entsprechende Operationen zwar stationär gemäß DRG-System offensichtlich kostendeckend durchführen zu können, was aber aufgrund von spezifisch ausgerichteten Krankenhausstrukturen ambulant aufgrund einer ggf. nicht auskömmlichen Entgeltpauschale im EBM nicht mehr möglich erscheint. Es liegt auf der Hand, dass nicht auskömmlich vergütete Leistungen dauerhaft nicht erbracht werden können; dennoch ist aber gerade die umfassende Strabologie, ausgehend von dezidierter präoperativer Diagnostik bis über die spezialisiert durchgeführte Operation und hin zur spezifischen Nachkontrolle, ein Segment, welches nur verhältnismäßig wenige Ophthalmochirurgen in der an einer Klinik notwendigen Form beherrschen und/oder durchführen. Hinsichtlich einer solch spezialisierten Expertise scheinen es häufig Maximalversorger und/oder große OP-Zentren zu sein, die diese Leistungen in entsprechender Anzahl und offensichtlich überwiegend stationär anbieten.

In der Tat sind beispielsweise nach der Erhebung des Verbandes der Universitätsklinika Deutschlands (VUD) deutschlandweit 6910 in die DRG C10 gruppierte Eingriffe im Jahr 2018 gezählt worden [19]. Die Bewertungsrelation (Casemix-Index [CMI]) wurde dabei mit 0,61 angegeben, was impliziert, dass bereits sehr häufig die untere Grenzverweildauer (UGV) unterschritten wurde, sodass stationär bereits ein deutlicher Entgeltabschlag von den Krankenhäusern hingenommen wurde (regulärer CMI 0,718 für die DRG C10B; regulärer CMI 0,661 für die DRG C10C) [10]. Nach Angaben des „Reimbursement Institutes“ wurden für das Jahr 2018 insgesamt etwa 7850 stationäre Fälle in der DRG C10B und C gezählt [16]. Zusätzlich ergaben sich offensichtlich auch in der hochpreisigen DRG C10A etwa 750 Fälle [16], die – verglichen mit unserem Kollektiv (DRG C10A lediglich 3‑mal vorhanden) – somit verhältnismäßig häufig abgerechnet wurden. Würde man nach diesen Daten nun von deutschlandweit insgesamt 8600 strabologischen stationär versorgten Fällen in 2018 ausgehen, dann scheinen über 80 % aller stationären Fälle von den Unikliniken versorgt worden zu sein, was aufzeigen würde, dass dieses Operationsspektrum in der Tat maßgeblich von Maximalversorgern als „Spezialsegment“ betreut wird und somit auch entsprechend „höhere“ Kosten generiert werden. Nach persönlicher Kommunikation geht der Berufsverband der Augenärzte (BVA) ebenfalls davon aus, dass chirurgische Strabologie nur selten in einem rein ambulanten Setting durchgeführt wird (persönliche Kommunikation 07.05.2020). Dieses würde dann auch nochmals die offensichtlich besondere Expertise, die sich durch die rein betriebswirtschaftlichen Personalkostenstrukturen (lediglich in Abhängigkeit der notwendigen Zeit für die erbrachte Leistung) allerdings nicht in adäquater Weise abbilden, unterstreichen.

Bezüglich des ambulanten Operierens wurden Krankenhäuser mit der Einführung des § 115b SGB V durch das Gesundheitsstrukturgesetz im Jahr 1992 zur Durchführung von ambulanten Operationen zugelassen [1]. Der Gesetzgeber beauftragte KBV, GKV und DKG damit, in einem dreiseitigen Vertrag (AOP-Vertrag) unter anderem einen Katalog der ambulanten Operationen (AOP-Katalog) zu vereinbaren [1]. Nach § 4 dieses Vertrages sind die Leistungen über den EBM [3] abzurechnen und nach § 7 die Krankenhäuser bei der Vergütung ambulanter Leistungen wie niedergelassene Fachärzte der entsprechenden Fachrichtung einzustufen [1]. Nach Stand 01.01.2019 des EBM [3] werden strabologische Operationen unter den Punkten 5‑10a.0 bis 5‑10k.8 ↔ abgerechnet. Nach dem EBM erhöht sich die Vergütung der chirurgischen Leistung zwar auch nach Schweregrad und teilweise auch nach Anzahl der operierten Muskeln, allerdings scheinen die Erlöse insgesamt relativ niedrig auszufallen. Folgende Entgelte unter vereinfachter und grober Betrachtung der Anzahl der operierten geraden Augenmuskeln werden aufgeführt: 1 Muskel – U2 – 159,86 €; 2 Muskeln – U3 – 233,92 €; 3 Muskeln – U3/U4 – 233,92 bis 313,13 €; 4 Muskeln – U5 – 416,85 € [3]. Zuzüglich der anästhesiologischen Leistungen kommt es zu Gesamtentgelten, die zwischen ca. 408,50 und 662,80 € bei ambulanter Leistungserbringung liegen (Daten aus Baden-Württemberg). Dezidierte Entgeltbetrachtungen sind über die Homepage der KBV [3] möglich.

Unsere Kostenträgerrechnungen für strabologische OPs an einer Universitäts-Augenklinik zeigen aber nun, dass hier durchschnittliche Kosten von 930,83 € für die reine OP-Leistung nach dezidiert betriebswirtschaftlich errechneten Faktoren anfallen, wobei Operationen bei kleinen Kindern und/oder mit mehr als 2 Muskeln bereits nochmals erheblich teurer sind (Abb. 4). Die Kosten der stationären Leistung sind dabei explizit nicht einbezogen. Es liegt im direkten Vergleich der reinen OP-Kosten gegenüber dem ambulanten Entgelt somit eine deutliche Unterfinanzierung (ungefähr Faktor 2) vor, wenn davon ausgegangen wird, dass die Personal-/und Raumstrukturen an der Uniklinik für den stationären und den ambulanten Bereich gleich sind. Dieses ist in der MHH für alle narkosebasierten Operationen in der Tat der Fall. Beispielhaft zeigen damit *n* = 10 Fälle (DRG C10C) aus dem Jahr 2019 in unserem Kollektiv, die nachträglich durch den MDK in den AOP-Bereich überführt wurden, dass sich die Gesamtrechnungssumme von 20.560,19 € um 15.656,16 € auf nur noch 4904,03 € reduziert hat, womit eine strabologische OP dieser Kategorie nur noch ca. 490 € erlöst und somit nicht mehr kostendeckend erbracht werden kann. Zusätzlich finden nach dieser Korrektur auch die stationären Kosten – die ja pro Fall vorhanden waren (da der Patient ja stationär versorgt wurde!) – keine Berücksichtigung, sodass insgesamt bei erbrachter Leistung zu niedrige Erlöse und gleichzeitig zu hohe Kosten vorliegen.

Hält man sich unter den genannten Gesichtspunkten vor Augen, dass gerade größere Krankenhäuser diejenigen Leistungserbringer sind, welche die offensichtlich doch recht spezialisierte chirurgische Strabologie (inklusive prä- und postoperativer zum Teil erheblich aufwendiger Versorgung) erbringen und somit diese Leistungen auch vorhalten müssen, so sollte man der traditionell häufig in Krankenhäusern vielleicht schlechter für ambulante Leistungen ausgerichteten Infrastruktur Rechnung tragen, dass medizinische Leistungen teurer sind. Dazu kommt weiterhin der Umstand, dass gerade in diesen Häusern Studierende, Ärzte und Chirurgen aus-/weiter- und fortgebildet werden, was z. B. für das Fach Augenheilkunde bedeuten kann, dass 2 (Fach‑)Ärzte „nur“ *eine* Leistung erbringen (chirurgische Weiterbildung findet häufig erst nach der Facharztweiterbildung statt), die in einer Fachklinik regelhaft nur ein versierter Facharzt erbringen würde. Beispielhaft ist Letzteres im Rahmen von Kataraktoperationen der Fall, die üblicherweise sehr häufig und zumeist ambulant hoch spezialisiert in Fachzentren durchgeführt werden. Für Strabismusoperationen scheint dieses aber nicht unbedingt zu gelten, da bei Muskeloperationen – im Gegensatz zu Kataraktoperationen – zumeist auch ein Assistent benötigt wird und dieses Segment offensichtlich in vielerlei Hinsicht sehr speziell und definitiv auch mit deutlich erhöhtem perioperativem Aufwand verbunden ist. So gesehen, könnte natürlich diskutiert werden, dass zur Leistungserbringung hier eine besondere häufig an Kliniken vorhandene Spezialisierung vorliegt, welche entsprechend auch höher honoriert werden dürfte. Eine solche bessere Honorierung ist im Krankenhauswesen allerdings nicht regelhaft vorgesehen und gerade ambulant – wie oben bereits beschrieben – mit einer Gleichstellung bezüglich der Entgelte versehen [1], ohne dass natürlich die Kostenstrukturen von Krankenhäusern und Praxen miteinander vergleichbar wären.

Bezüglich der in unserer Erhebung dargestellten Kostenergebnisse muss angemerkt werden, dass die realen Kosten für die gleiche Leistung in anderen Häusern je nach baulicher und personeller Infrastruktur, den Overheads, dem Patientenaufkommen, etc. eben auch anders ausfallen (können). Auch unterliegen unsere Daten hinsichtlich der Erlösseite für ambulante Operationen der Limitation, dass unsere Kostenträgerrechnung nicht in einem ambulanten OP-Setting durchgeführt wurde, sondern für einen Zentral-OP für regulär stationäre Operationen, die einem anderen Kostendruck unterliegen. Andererseits muss aber auch der Umstand betrachtet werden, dass innerhalb der Kostenträgerrechnung lediglich die entsprechenden Kosten auf die reine OP-/Anwesenheitszeit in Minuten dargestellt werden, nicht aber definitiv häufig auch vorhandene Leerzeiten, z. B. zwischen einzelnen Operationen, mitgerechnet werden. Da aber ja auch zu diesen Zeiten insbesondere Personal- und Raumkosten (Vorhaltekosten) anfallen, muss davon ausgegangen werden, dass die in unserer Aufstellung errechneten Kosten eher sogar noch zu niedrig kalkuliert sind. Weiterhin wären auch ein Vergleich der prä- und postoperativen Diagnostik und der Nachbehandlung sowie deren Kostenstrukturen interessant – entsprechende Daten möchten wir zukünftig in dieser Hinsicht noch erfassen und auswerten.

Prinzipiell zeigt diese Auswertung beispielhaft für eine Uniklinik, dass Kosten für Strabismusoperationen deutlich höher ausfallen als die Entgelte, die aktuell über ambulantes Operieren erlöst werden können, sodass diese Leistungen unter betriebswirtschaftlichen Bedingungen definitiv nicht regelhaft angeboten werden können. So gesehen, ist gerade die hoch spezialisierte Augenheilkunde aufgrund der heutzutage stationär niedrigen Liegezeiten leider ein gutes Beispiel dafür, dass diese medizinisch ja eigentlich für alle Beteiligten positive Entwicklung kürzerer Liegezeiten abrechnungstechnisch noch weiter „ausgenutzt“ wird, stationär erbrachte Leistungen im Nachhinein zu „ambulantisieren“, was somit zu einer nicht mehr sachgerechten Finanzierung führt. Beispielhaft zeigt hier nun die chirurgische Strabologie das Dilemma der Krankenhäuser auf, ambulante Leistungen weiter forcieren zu müssen, diese aber nicht adäquat vergütet zu bekommen. Hier muss ein Umdenken und auch z. B., wie hier geschehen, eine auskömmliche Kostenberechnung unter objektiv nachvollziehbaren und betriebswirtschaftlichen Aspekten stattfinden, bei der auch Weiter- und Fortbildung – gerade an Unikliniken – berücksichtigt werden müssen. Ebenfalls muss aber auch erkannt werden, dass sowohl im ambulanten Hochschulambulanzbereich als auch im niedergelassenen Bereich die Quartalspauschalen oder andere Budgetierungen definitiv viel zu niedrig angesetzt sind, als dass die sehr aufwendigen ambulanten perioperativen Leistungen bei Schiel-OPs und auch insgesamt die eher komplexen Leistungen in der Strabologie adäquat finanziert wären. Diese massive Unterfinanzierung im ambulanten Bereich mag durchaus auch der Grund dafür sein, dass offensichtlich überwiegend nur die Maximalversorger diese Leistungen überhaupt noch anbieten, da sie dieses durch querfinanzierte Mischkalkulationen zumindest bisher gerade noch ermöglichen konnten. Allerdings erscheint – im Gegensatz zur Weiter-/und Fortbildung bei Kataraktoperationen und ggf. Vitrektomien – eine chirurgische Fortbildung in dem strabologischen Segment für Augenärzte eher unattraktiv, da eben die gesamte Strabologie extern und ambulant nicht kostendeckend und nur mit viel Aufwand erbracht werden kann. Wäre dieser Bereich besser honoriert, würde es die Attraktivität dieses spannenden Faches wahrscheinlich enorm steigern, es könnten dann vielleicht mehr Behandlungen und notwendige Operationen auch im ambulanten Setting und unabhängig von Maximalversorgern durchgeführt werden, was vielen Patienten mit entsprechenden Erkrankungen – ggf. ohne lange Wartezeiten und wohnortnah – die Lebensqualität enorm verbessern würde. Es ist den Autoren ein besonderes Anliegen, dass diese Problematik erkannt wird und Honorarstrukturen für spezialisierte Bereiche deutlich verbessert werden müssen, um auch langfristig noch Ärzte zu haben, die in diesen besonderen Bereichen der Augenheilkunde spezialisiert im Sinne der Patienten agieren mögen.
